# Rational Design of 2D/3D Bi_2_O_2_Se–CNT Hybrid Architectures for Synergistic Lithium Storage

**DOI:** 10.3390/molecules30081685

**Published:** 2025-04-09

**Authors:** Duqiang Xin, Yue Zhang, Yeming He, Jiao Liu, Wenyuan Duan, Guoxiu Han, Qi Zhang, Yuming Yang

**Affiliations:** 1 School of Electronic Information, Xijing University, Xi’an 710123, China; 2 Shaanxi Engineering Research Center of Controllable Neutron Source, Xijing University, Xi’an 710123, China; 3 School of Foreign Languages, Xijing University, Xi’an 710123, China

**Keywords:** Bi_2_O_2_Se, 2D layered materials, CNT networks, structural stability, lithium-ion batteries

## Abstract

The development of advanced anode materials with high capacity and structural stability addressing the limitations of conventional graphite anodes in theoretical capacity (372 mA h g^−1^) and severe volume expansion remains a critical challenge for lithium-ion batteries (LIBs). Herein, we propose a structural engineering strategy through high-temperature calcination to construct 2D layered Bi_2_O_2_Se integrated with optimized 3D carbon nanotube (CNT) frameworks (Bi_2_O_2_Se–CNT-x). Comprehensive characterization (XRD, Raman, FESEM, XPS) verifies the high crystallinity of Bi_2_O_2_Se and the successful establishment of 3D conductive networks through interfacial coupling with CNTs. Electrochemical evaluation demonstrates that the optimized Bi_2_O_2_Se–CNT-2 composite delivers a remarkable initial discharge capacity of 1544.7 mA h g^−1^ at 0.1 A g^−1^, significantly outperforming pristine Bi_2_O_2_Se (124.3 mA h g^−1^). Notably, it maintains superior rate capability (405.0 mA h g^−1^ at 2 A g^−1^, 35.2% capacity retention) and cycling stability (74.8% capacity retention after 250 cycles), attributed to the synergistic effects between 2D Bi_2_O_2_Se lamellae and the conductive CNT matrix. The 3D CNT network facilitates rapid electron transport while mitigating volume fluctuations, whereas the layered Bi_2_O_2_Se enables a hybrid storage mechanism combining intercalation, conversion, and alloying reactions. This work broadens the application horizons of 2D layered materials in energy storage systems.

## 1. Introduction

Secondary batteries, characterized by their high energy conversion efficiency, superior energy/power density, and flexible installation, have been extensively utilized across multiple domains [1]. Among commercial secondary batteries, lead–acid batteries demonstrate high stability and cost-effectiveness, primarily serving as starting batteries in automobiles and electric motorcycles. However, their environmental impact remains a critical concern due to the high toxicity of lead and associated pollution from waste disposal [2]. Nickel–cadmium batteries exhibit exceptional rate performance, yet their practical applications are constrained by severe memory effects that significantly shorten service life [3]. Nickel–metal hydride batteries feature remarkable low-temperature performance, though their widespread adoption has been hindered by elevated production costs [4]. Since their emergence in 1991, lithium-ion batteries (LIBs) have dominated energy storage markets owing to their unparalleled advantages: high energy density, long cycle life, rapid charging capability, absence of memory effect, low self-discharge rate, and environmental benignity. These merits have enabled their pervasive implementation in diverse fields, including 3C digital devices, electric vehicles, energy storage systems, aerospace technologies, and medical equipment [5,6]. However, the ever-increasing demand for high-energy-density lithium-ion batteries has spurred intensive research into advanced anode materials, aiming to overcome the inherent limitations of conventional graphite anodes, such as low theoretical capacity (372 mA h g^−1^) and severe volume expansion during cycling [7,8,9]. While emerging candidates like silicon [10], transition metal oxides [11,12], and sulfides [13,14,15] exhibit higher capacities, their practical applications are hindered by intrinsic drawbacks, including drastic volume changes, sluggish reaction kinetics, and poor cyclability. Consequently, exploring novel anode materials with balanced capacity, structural stability, and conductivity remains a pivotal, yet challenging task for next-generation LIBs.

Recent advancements highlight the potential of two-dimensional (2D) layered materials as promising alternatives due to their unique ion transport channels and tunable interlayer spacing [16,17,18]. Among them, bismuth oxyselenide (Bi_2_O_2_Se), an emerging 2D layered semiconductor material with a distinctive non-electroneutral structure featuring alternating [Bi_2_O_2_]^2+^ and [Se]^2−^ layers, has garnered significant attention in electronic devices such as field-effect transistors [19,20,21,22]. Its large interlayer spacing (0.61 nm) and high carrier mobility endow it with substantial theoretical capacity potential for lithium storage applications [23,24,25]. Wu et al. demonstrated the synthesis of layered Bi_2_O_2_Se via a solid-state reaction in the evacuated quartz glass ampoule, followed by the fabrication of Bi_2_O_2_Se–graphite composites through ball-milling. When evaluated as an LIBs anode, the composite delivered reversible capacities of 530 mAh g^−1^ at 0.1 A g^−1^ and 214 mAh g^−1^ at 5 A g^−1^, though limited by unsatisfactory cycling stability. Synchrotron-based X-ray absorption spectroscopy (XAS) elucidated a multi-step lithium storage mechanism involving insertion–conversion–alloying processes [26]. This pioneering work establishes Bi_2_O_2_Se as a novel 2D anode material while proposing structural optimization strategies to mitigate selenide shuttling and enhance electrochemical performance. Despite these advancements, the achievable specific capacity and cycling stability remain suboptimal. These challenges underscore the necessity and innovation of developing carbon-composited Bi_2_O_2_Se architectures through rational structural design to advance lithium storage capabilities, thereby addressing both fundamental mechanistic understanding and practical application requirements.

In this work, a rational structural engineering strategy is proposed to construct a 2D/3D Bi_2_O_2_Se–carbon nanotube (CNT) hybrid architecture (Bi_2_O_2_Se–CNT-x) via high-temperature calcination. The interfacial coupling between 2D Bi_2_O_2_Se lamellae and a 3D interconnected CNT network establishes an efficient conductive framework that simultaneously enhances charge transfer kinetics and accommodates volume variations during cycling, while preserving structural integrity. The layered Bi_2_O_2_Se enables multi-step lithium storage mechanisms involving intercalation, conversion, and alloying reactions to ensure the lithium storage capacity of the hybrid architecture. Systematic compositional optimization achieves an optimal balance between active material loading and conductive pathways, with the optimized Bi_2_O_2_Se–CNT-2 composite demonstrating exceptional electrochemical performance: a high initial discharge capacity of 1544.7 mA h g^−1^ at 0.1 A g^−1^, remarkable rate capability (405.0 mA h g^−1^ at 2 A g^−1^), and durable cyclability with 74.8% capacity retention after 250 cycles. This work provides a universal strategy for advanced 2D/3D hybrid electrodes and deepens the understanding of multi-mechanistic lithium storage in layered materials, demonstrating practical potential for next-generation energy storage systems.

## 2. Results and Discussion

**Figure 1:**
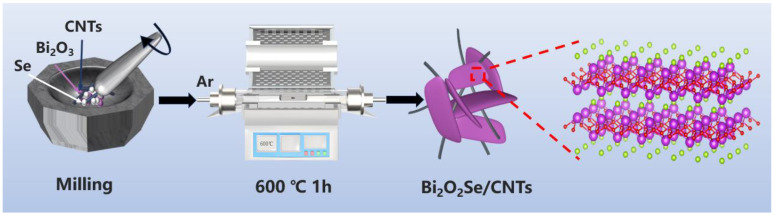
Schematic illustration of the synthesis of Bi_2_O_2_Se–CNT-x composites.

Multi-walled carbon nanotubes (MWCNTs), composed of multiple concentric cylindrical graphene layers (typically 2–20 layers), demonstrate superior mechanical toughness, stable electrical conductivity, enhanced thermal stability, and lower production costs compared to single-walled carbon nanotubes (SWCNTs) formed by a singular rolled graphene sheet. These advantages motivated the strategic integration of MWCNTs with Bi_2_O_2_Se in this study, thereby synergistically improving both electrical conduction performance and structural robustness in the resultant composites. For simplicity, MWCNTs are abbreviated as CNTs. As shown in Figure 1, the Bi_2_O_2_Se–CNT-x composites were synthesized through a high-temperature calcination strategy, wherein 2D layered Bi_2_O_2_Se lamellae were integrated with a 3D acid-treated CNT conductive framework. During the synthesis, selenium atoms derived from the precursor powder selectively substituted oxygen atoms in the Bi_2_O_3_ matrix, inducing the growth of Bi_2_O_2_Se lamellae with a layered architecture that facilitates rapid Li^+^ diffusion pathways and exposes abundant electrochemically active sites. The pre-functionalized CNT network serves as a multifunctional scaffold, synergistically enhancing the composite’s electrical conductivity through interconnected percolation pathways while simultaneously alleviating mechanical stress caused by the repetitive volume expansion–contraction of Bi_2_O_2_Se during cycling. This unique configuration leverages the structural advantages of both components, collectively optimizing the composite’s electrochemical performance [23,27].

**Figure 2:**
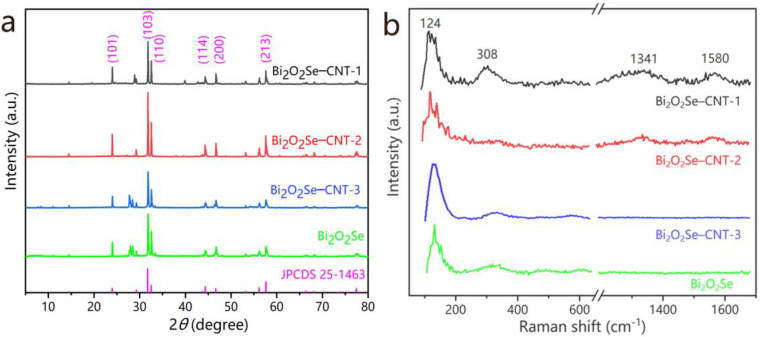
(**a**) XRD patterns and (**b**) Raman spectra of pristine Bi_2_O_2_Se and Bi_2_O_2_Se–CNT composites.

The crystal structures of Bi_2_O_2_Se and its CNT composites were characterized by XRD. As shown in Figure 2a, pristine Bi_2_O_2_Se exhibits well-defined diffraction peaks at 2*θ* ≈ 24.0°, 31.7°, 32.5°, 46.6°, and 57.6°, which correspond to the (101), (103), (110), (200), and (213) planes, respectively, matching the tetragonal phase of Bi_2_O_2_Se (JCPDS 25-1463). The sharp peaks indicate the high crystallinity and phase purity of the synthesized material. For the Bi_2_O_2_Se–CNT composites with different CNT content, the characteristic peaks of Bi_2_O_2_Se remain prominent, confirming the host crystal structure during composite formation. No carbon phase peaks can be found in the XRD pattern, suggesting the acid-treated CNTs contained in the composites are amorphous carbon without regular crystal structure. Moreover, there is no significant peak broadening and peak shifts, indicating minimal lattice distortion and weak interfacial interaction between Bi_2_O_2_Se and CNTs. These structural features are critical for optimizing charge transport properties in energy storage applications, as coherent interfaces and preserved crystallinity enhance electronic conductivity and ion diffusion kinetics.

Figure 2b shows the Raman spectra of pristine Bi_2_O_2_Se and Bi_2_O_2_Se–CNT composites. All samples exhibit distinct peaks at 124 and 308 cm^−1^, corresponding to the vibrational modes of Bi_2_O_2_Se, respectively, which are attributed to the in-plane and out-of-plane vibrations of the [Bi_2_O_2_]^2+^ layers and Se atomic motions in the crystal lattice [24,25,28]. These features also confirm the structural integrity of Bi_2_O_2_Se in both pristine and composite structures. Notably, the Bi_2_O_2_Se–CNT-1 composite demonstrates additional characteristic bands at 1347 cm^−1^ (D-band) and 1580 cm^−1^ (G-band), which decline with decreasing CNT content and disappear in Bi_2_O_2_Se–CNT-3, with the lowest CNT content, and in pure Bi_2_O_2_Se. The D-band arises from structural defects or disordered sp^3^ carbon in the CNTs, while the G-band corresponds to the in-plane vibration of sp^2^-bonded carbon atoms in the graphitic lattice [17]. The *I*_D_/*I*_G_ ratios in Bi_2_O_2_Se–CNT-1 and Bi_2_O_2_Se–CNT-2 were 2.5 and 1.9, respectively, indicating that acid-treated CNTs had a large number of defects and were highly disordered. The presence of these D and G peaks confirms the successful integration of CNTs into the composite, which is advantageous for improving the electrical conductivity.

**Figure 3:**
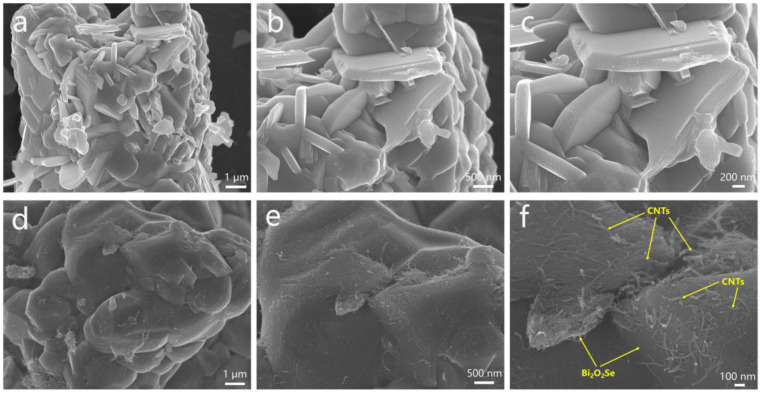
FESEM images of (**a**–**c**) Bi_2_O_2_Se and (**d**–**f**) Bi_2_O_2_Se–CNT-2 at different magnifications.

Figure 3 presents the FESEM images of Bi_2_O_2_Se and its composite Bi_2_O_2_Se–CNT-2. The pristine Bi_2_O_2_Se exhibits a stacked-block morphology with individual blocks measuring 1–2 μm in length and 200–300 nm in thickness. High-magnification imaging (Figure 3c) reveals distinct 2D layered structural features. This layered architecture facilitates efficient Li^+^ intercalation–deintercalation during electrochemical processes. Notably, abundant interstitial pores between the stacked blocks create additional Li^+^ storage spaces, which may enhance specific capacity. In the Bi_2_O_2_Se–CNT-2 composite (Figure 3d–f), the Bi_2_O_2_Se component retains its original block-stacking morphology while being intricately interconnected by an entangled network of CNTs with uniform diameters of approximately 15 nm. These CNTs not only bridge adjacent Bi_2_O_2_Se blocks but also establish 3D electronically conductive pathways throughout the composite. This structural configuration is anticipated to simultaneously reduce Li^+^ diffusion distances and improve overall electrical conductivity. Figure S1 shows the FESEM comparison of Bi_2_O_2_Se–CNT composites with different CNT content at different magnifications, which further demonstrates the composition-dependent morphology evolution, where increased CNT loading results in denser nanotube entanglement around Bi_2_O_2_Se blocks.

**Figure 4:**
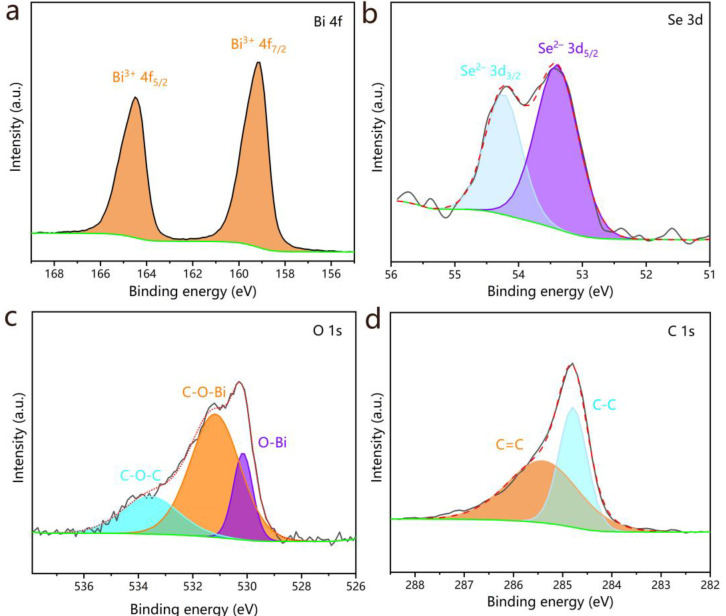
High-resolution XPS spectra of (**a**) Bi 4f, (**b**) Se 3d, (**c**) O 1s, and (**d**) C 1s for Bi_2_O_2_Se–CNT-2.

The elemental composition and chemical states of Bi_2_O_2_Se–CNT-2 were investigated by XPS. In Figure S2, the survey spectra verify the concomitance of Bi, Se, O, and C elements. As depicted in Figure 4a, the Bi 4f high-resolution spectrum exhibits two well-resolved spin-orbit peaks located at 164.5 eV (Bi^3+^ 4f_5/2_) and 159.1 eV (Bi^3+^ 4f_7/2_) [23,25,28]. The 5.4 eV splitting energy between these peaks aligns with standard values for Bi^3+^ in Bi_2_O_2_Se, confirming the successful formation of the bismuth oxyselenide phase. The Se 3d region (Figure 4b) presents a doublet at 54.2 eV (Se^2−^ 3d_3/2_) and 53.4 eV (Se^2−^ 3d_5/2_) with a spin-orbit splitting of 0.8 eV, consistent with selenide species in Bi_2_O_2_Se [24,28]. Deconvolution of the O 1s spectrum (Figure 4c) reveals three distinct contributions: the peak at 530.2 eV corresponding to lattice oxygen in O-Bi bonds, and the two peaks located at 531.2 and 533.6 eV were conclusively assigned to C-O-Bi and C-O-C bonding configurations, respectively, thereby demonstrating effective interfacial coupling between Bi_2_O_2_Se and CNTs [23]. The C 1s spectrum (Figure 4d) resolves into two peaks at 284.8 eV (C-C) and 285.4 eV (C=C) [20].

**Figure 5:**
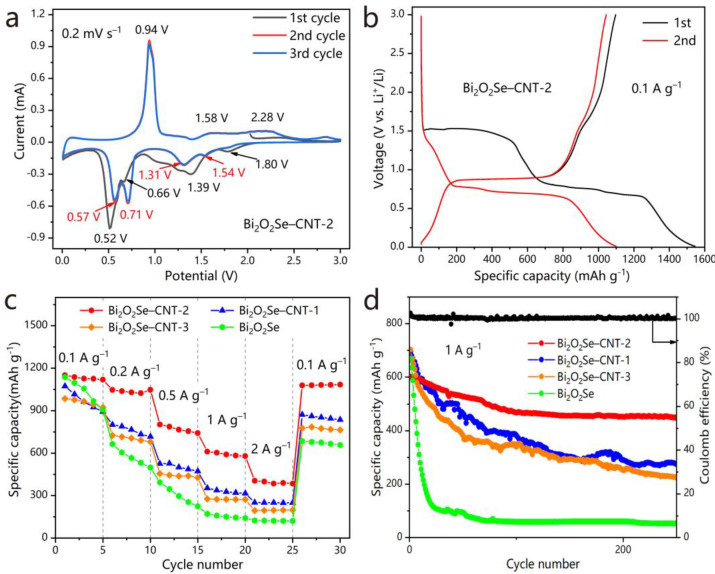
(**a**) CV curves of Bi_2_O_2_Se–CNT-2 for the initial three cycles at 0.2 mV s^−1^. (**b**) Galvanostatic charge–discharge curves of Bi_2_O_2_Se–CNT-2 at 0.1 A g^−1^ for the initial two cycles. Comparison of the rate performance (**c**) and cycle performance (**d**) of Bi_2_O_2_Se and three of its composites with varying CNTs.

The electrochemical lithium storage behavior of Bi_2_O_2_Se–CNT-2 was investigated via CV in the range of 0–3.0 V (versus Li/Li^+^) at a scan rate of 0.2 mV s^−1^ (Figure 5a). During the initial cathodic scan, four distinct reduction peaks were observed, corresponding to sequential Li^+^ intercalation, conversion, and alloying reactions [23,26]. Specifically, the peak at 1.80 V is attributed to the intercalation process, forming Li_x_Bi_2_O_2_Se (Equation (1)) [26,29,30,31]. The subsequent peak at 1.39 V signifies a conversion reaction, yielding metallic Bi, Li_2_O, and Li_2_Se (Equation (2)) [26,32]. The two lower-potential peaks at 0.66 and 0.52 V are assigned to the stepwise alloying reactions, generating LiBi and Li_3_Bi, respectively (Equations (3) and (4)) [23,26,32,33,34]. In the first anodic scan, a dealloying reaction occurs at 0.94 V, characterized by the oxidation of Li_3_Bi to Bi (Equation (5)) [29,30,31,32,33,34], followed by a broad oxidation peak spanning 1.58–2.28 V, which corresponds to the reconversion of Bi, Li_2_O, and Li_2_Se back to Bi_2_O_2_Se (Equation (6)) [30,33]. During the second and third cycles, the cathode peak at 1.39 V splits into two peaks, 1.54 V and 1.31 V, corresponding to the separated generation of Li_2_Se and Li_2_O [26,31]. The cathode peaks at 0.66 V and 0.52 V were shifted to higher potentials (0.71 and 0.57 V), likely due to the formation of SEI layer or reduced polarization during the first cycle [35]. In contrast, the anodic peaks remained unchanged across cycles. Remarkably, the curves of the second and third cycles exhibit substantial overlap, demonstrating the excellent electrochemical reversibility of the Bi_2_O_2_Se–CNT-2 anode material. The above results elucidate a hybrid lithium storage mechanism in Bi_2_O_2_Se–CNT-2, synergistically combining intercalation, conversion, and alloying processes. The intercalation enhances structural stability by buffering volume changes, while the conversion and alloying reactions contribute to high theoretical capacity. Such a multi-mechanism coupling strategy provides a promising pathway to balance capacity, kinetics, and cycle stability in advanced LIBS electrodes.

For comparative analysis, the CV curves of pure Bi_2_O_2_Se electrode are supplemented in Figure S3. As can be seen in Figure 5a and Figure S3, Both Bi_2_O_2_Se–CNT-2 and pure Bi_2_O_2_Se electrodes display nearly identical peak positions and quantities, though the Bi_2_O_2_Se–CNT-2 exhibits significantly reduced peak intensities compared to pure Bi_2_O_2_Se, attributable to the incorporation of CNTs. Crucially, the CNTs framework provides structural stabilization, as evidenced by the excellent overlap of successive CV cycles for Bi_2_O_2_Se–CNT-2. In contrast, pure Bi_2_O_2_Se demonstrates progressive peak intensity attenuation during cycling, highlighting the critical role of CNTs in enhancing electrochemical stability.

(1) $${\mathrm{Bi}}_{2}O_{2}\mathrm{Se}+xLi^{+}+xe^{-}\to Li_{x}{\mathrm{Bi}}_{2}O_{2}\mathrm{Se}$$

(2) $$Li_{x}{\mathrm{Bi}}_{2}O_{2}\mathrm{Se}+\left(6-x\right)Li^{+}+\left(6-x\right)e^{-}\to 2\mathrm{Bi}+2Li_{2}O+Li_{2}\mathrm{Se}$$

(3) $$\mathrm{Bi}+Li^{+}+e^{-}\to \mathrm{LiBi}$$

(4) $$\mathrm{LiBi}+2Li^{+}+2e^{-}\to Li_{3}\mathrm{Bi}$$

(5) $$Li_{3}\mathrm{Bi}\;\to \mathrm{Bi}+3Li^{+}+3e^{-}$$

(6) $$2\mathrm{Bi}+2Li_{2}O+Li_{2}\mathrm{Se}\;\to {\mathrm{Bi}}_{2}O_{2}\mathrm{Se}+6Li^{+}+6e^{-}$$

Figure 5b displays the galvanostatic charge–discharge profiles of Bi_2_O_2_Se–CNT-2 anode during the initial two cycles at 0.1 A g^−1^ within the voltage window of 0.01–3.0 V (vs. Li^+^/Li). During the initial discharge process, two pronounced plateaus emerge at 1.4–1.5 V and 0.6–0.8 V. The first plateau, based on the CV results, is related to Li^+^ intercalation. But on the second cycle, this plateau is much smaller and aligns with the attenuated redox peaks in the CV curves (Figure 5a), indicating reduced intercalation activity during subsequent cycles. This behavior is attributed to structural reorganization or partial irreversibility of the intercalation process after the initial cycle [26]. While intercalation contributes dominantly to the first-cycle capacity, the subsequent cycling is primarily governed by conversion and alloying reactions, as evidenced by the persistent redox peaks associated with these mechanisms in the CV profiles. The narrowing of the intercalation plateau does not imply its complete absence, but rather highlights a shift in the dominant charge storage mechanisms, consistent with the electrochemical behavior of bismuth-based chalcogenides [29,32,34]. The subsequent charge process exhibits a well-defined plateau near 0.9 V. Crucially, ultrawide voltage plateaus persist even at 2 A g^−1^ (Figure S4), which are consistent with CV curves and also suggest a stable interfacial reaction kinetics and minimized polarization.

The Bi_2_O_2_Se–CNT-2 anode delivers an impressive initial discharge capacity of 1544.7 mAh g^−1^ and a charge capacity of 1097.4 mAh g^−1^ at 0.1 A g^−1^, yielding a coulombic efficiency (CE) of 71.1%. The capacity loss primarily originates from the inevitable formation of a solid–electrolyte interphase (SEI), incomplete conversion reactions, and partial electrolyte decomposition [36].

Figure 5c displays the rate performance of Bi_2_O_2_Se and three of its composites with varying CNTs. Compared to pristine Bi_2_O_2_Se, the Bi_2_O_2_Se–CNT composites demonstrated significantly improved electrochemical kinetics, attributed to the synergistic effects between conductive CNT networks and the Bi_2_O_2_Se matrix. In particular, the Bi_2_O_2_Se–CNT-2 anode delivered a discharge specific capacity of 405.0 mAh g^−1^ at 2 A g^−1^, far surpassing the values of pristine Bi_2_O_2_Se (124.3 mAh g^−1^), Bi_2_O_2_Se–CNT-1 (251.0 mAh g^−1^), and Bi_2_O_2_Se–CNT-3 (195.0 mAh g^−1^). Notably, upon reverting to 0.1 A g^−1^ after high-rate cycling, Bi_2_O_2_Se–CNT-2 retained 93.7% of its initial specific capacity (1078.6 vs. 1150.8 mAh g^−1^), demonstrating exceptional structural reversibility. The CNT content profoundly influenced the rate performance. While insufficient CNTs (Bi_2_O_2_Se–CNT-3) failed to establish a continuous conductive framework, excessive CNTs (Bi_2_O_2_Se–CNT-1) likely reduced the effective loading of active Bi_2_O_2_Se. In contrast, Bi_2_O_2_Se–CNT-2 with moderate CNT loading achieved optimal balance: the intertwined CNTs not only enhanced electron transport but also buffered volume expansion during cycling, thereby maintaining electrode integrity [17]. This was further corroborated by its superior capacity retention of 35.2% at 2 A g^−1^ relative to 0.1 A g^−1^, outperforming Bi_2_O_2_Se–CNT-1 (23.4%), Bi_2_O_2_Se–CNT-3 (19.8%), and pristine Bi_2_O_2_Se (10.9%). Notably, the theoretical specific capacity of Bi_2_O_2_Se as LIBs anode is calculated to be 608 mAh g^−1^ based on the conversion reaction mechanism, which assumes the participation of Bi, O, and Se in lithium storage reactions, forming Li_3_Bi, Li_2_O, and Li_2_Se, respectively. However, the experimentally measured initial specific capacity of pure Bi_2_O_2_Se reaches 1100 mAh g^−1^ at 0.1 A g^−1^, significantly exceeding the theoretical value. The primary reasons for this discrepancy are the synergistic integration of multi-mechanistic lithium storage and structural advantages inherent to its nanoengineered design. While the theoretical capacity is derived solely from conversion reactions, the practical electrochemical process involves intercalation within the layered structure, alloying, and surface–interface capacitive effects, collectively surpassing the single-mechanism assumption. Moreover, the material’s nanosheet morphology, interlayer voids, and defect-rich architecture also provide additional lithium adsorption sites and increases lithium storage capacity.

The cycle stabilities of Bi_2_O_2_Se-based anodes were investigated at 1 A g^−1^ (Figure 5d). While pristine Bi_2_O_2_Se suffered catastrophic capacity decay (from 669.9 to 52.9 mAh g^−1^ within 25 cycles), all CNT-incorporated composites exhibited markedly improved cycling stability. In particular, the Bi_2_O_2_Se–CNT-2 retained 450.4 mAh g^−1^ (74.8% capacity retention) after 250 cycles, significantly outperforming Bi_2_O_2_Se–CNT-1 (39.4%) and Bi_2_O_2_Se–CNT-3 (31.9%). This result further proves that suitable CNT content is crucial to improve the lithium storage performance of Bi_2_O_2_Se–CNT composites. Table S1 present a comparative analysis of cycle performance between our Bi_2_O_2_Se–CNT-2 composite and other Bi_2_O_2_Se- or Bi_2_Se_3_-based anode materials [26,29,30,31,32,33,34,35,37]. In this comparison, our Bi_2_O_2_Se–CNT-2 outperforms many reported materials owing to the layered structure and stable support from CNT networks, showing promising application prospects.

Further post-cycling characterization of both Bi_2_O_2_Se–CNT-2 composite and pure Bi_2_O_2_Se electrodes was conducted after 250 cycles through FESEM. As illustrated in Figure S5, the cycled Bi_2_O_2_Se–CNT-2 electrode retained its intrinsic layered bulk stacking structure, demonstrating exceptional structural integrity owing to the effective mechanical support from CNTs. Notably, the electrode surface exhibited abundant residual nanoparticles derived from electrochemical reactions, while the presence of a continuous SEI layer partially obscured the detailed surface morphology. In contrast, the pure Bi_2_O_2_Se electrode displayed similar surface coverage by SEI and reaction byproducts, but suffered from significant structural degradation. Comparative analysis revealed that the original large bulk domains in pristine Bi_2_O_2_Se fragmented into smaller debris after prolonged cycling, indicative of irreversible structural collapse induced by repeated lithiation–delithiation stress. This structural instability aligns with its inferior cycling performance. The distinct morphological evolution between composite and pristine electrodes underscores the critical role of CNT incorporation in mitigating structural pulverization through enhanced stress dissipation and framework stabilization.

**Figure 6:**
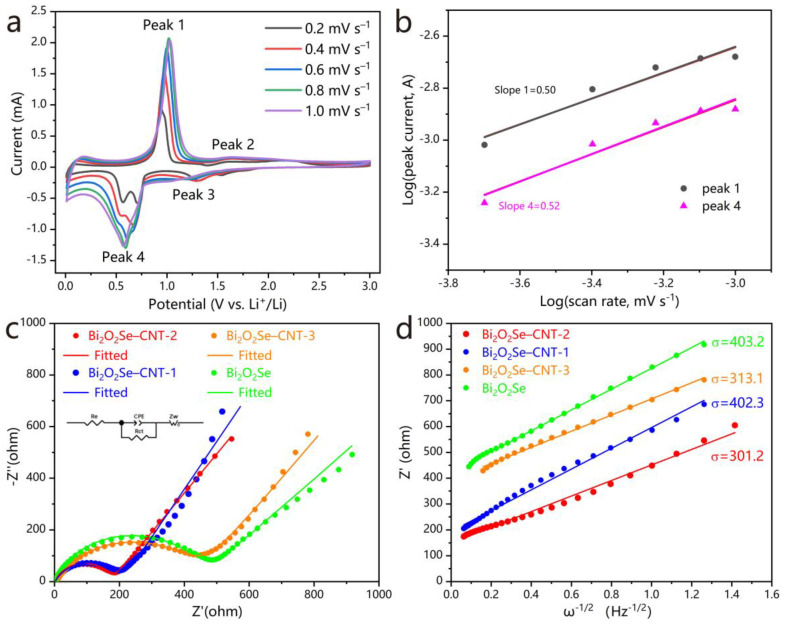
(**a**) CV curves of Bi_2_O_2_Se–CNT-2 for LIBs at scan rates from 0.2 mV s^−1^ to 1.0 mV s^−1^. (**b**) Plots of log(*i*) versus log(*v*) and their fitting values. (**c**) Nyquist plots of Bi_2_O_2_Se and three of its composites with varying CNT content. The inset is the equivalent circuit model. (**d**) Relation curves between Z` and *ω*^−1/2^.

The electrochemical kinetics of Bi_2_O_2_Se–CNT-2 anode were systematically investigated through CV at scan rates ranging from 0.2 to 1.0 mV s^−1^ (Figure 6a). Two prominent redox couples were identified: a dominant pair at 1.0 V (anodic) and 0.6 V (cathodic), accompanied by two minor peaks at 1.6 V (anodic) and 1.3 V (cathodic). Notably, peak current intensities exhibited a progressive enhancement with increasing scan rates, while anodic and cathodic peaks underwent slight potential shifts toward positive and negative directions, respectively, attributable to electrode polarization effects [38,39]. Crucially, the preserved CV profile symmetry across scan rates implies highly reversible redox processes at the electrode–electrolyte interface.

To deconvolute the charge storage mechanisms, the current response was analyzed using Equations (7) and (8) [38,39]:

*i* = *av*^*b*^ (7)

log*i* = *b*log*v* + log*a* (8)

where *i* denotes peak current, *v* represents scan rate, and *a* and *b* are constants. In Figure 6b, the derived *b*-values for dominant redox pairs (0.50 and 0.52) demonstrate proximity to the theoretical threshold of 0.5 for diffusion-controlled processes while deviating significantly from the capacitive limit (*b* = 1). The observed kinetic behavior aligns with the material’s layered structure, which facilitates intercalation-dominated lithium storage while maintaining structural integrity during repeated redox reactions.

To elucidate the enhanced electrochemical performance of the Bi_2_O_2_Se–CNT-2 anode, EIS analysis was performed on pristine Bi_2_O_2_Se and its composites before each cycle (Figure 6c). The Nyquist plots were quantitatively analyzed using ZView software (3.1.) with an equivalent circuit model (inset, Figure 6c), revealing critical interfacial charge transfer characteristics. Notably, the Bi_2_O_2_Se–CNT-2 composite demonstrates a substantially reduced charge-transfer resistance (*R*_ct_ = 172.2 Ω) compared to Bi_2_O_2_Se–CNT-1 (206.5 Ω), Bi_2_O_2_Se–CNT-3 (428.7 Ω), and pristine Bi_2_O_2_Se (439.9 Ω), indicating optimized interfacial kinetics and enhanced ion transport efficiency. The impedance comparison of pristine Bi_2_O_2_Se and its composites before cycling and after the first cycle was shown in Figure S6. Notably, the cycled samples exhibited a significant increase in impedance due to the formation of the SEI layer. This phenomenon is manifested as distinct changes in the high-frequency semicircles of the Nyquist plots. Specifically, Bi_2_O_2_Se–CNT-1 and Bi_2_O_2_Se–CNT-2 show a marked enlargement of their original semicircles, while Bi_2_O_2_Se–CNT-3 and pure Bi_2_O_2_Se display a newly emerged semicircle in the high-frequency region, which directly corresponds to the interfacial impedance of the SEI layer.

The relationship between *Z*′ and *ω*^−1/2^ in the low-frequency region was plotted and fitted, and the results are shown in Figure 6d. From the fitting, the Warburg factor *σ* can be obtained and the diffusion coefficient (*D*) can be calculated using Equations (9) [38]:

(9) $$D=\frac{R^{2}T^{2}}{2F^{4}n^{4}A^{2}C^{2}\sigma^{2}}$$

where *R* is the universal gas constant (8.314 J·mol^−1^·K^−1^), *T* is the absolute temperature, *n* is the number of electrons transferred per reaction (assumed as 1 for Li^+^), *F* is the Faraday constant (96,485 C·mol^−1^), *A* is the electrode area (m^2^), and *C* is the Li^+^ concentration in the electrode material (mol·m^−3^). The calculated *D* values are summarized in Table S2. In the table, the Bi_2_O_2_Se–CNT-2 electrode exhibits the highest diffusion coefficient (1.98 × 10^−17^ m^2^ s^−1^) among the tested electrodes, indicating significantly improved Li^+^ diffusion kinetics within the material. The enhanced diffusion kinetics ensures superior cycling stability and rate performance of the Bi_2_O_2_Se–CNT-2 electrode, particularly under high current densities.

## 3. Materials and Methods

### 3.1. Functionalized Treatment of MWCNTs

Commercial MWCNTs (2.0 g) were subjected to ultrasonic dispersion in 100 mL of binary acid solution (HNO_3_:H_2_SO_4_ = 1:1, *v*/*v*) using a high-power probe sonicator. After continuous ultrasonic treatment for 2 h, the resulting dispersion was subsequently subjected to continuous reflux under vigorous stirring at 120 °C for 4 h. Then, the system was gradually cooled to ambient temperature. The functionalized MWCNTs were collected via vacuum filtration using a polytetrafluoroethylene membrane (0.22 μm pore size), followed by sequential rinsing cycles with deionized water until the pH was neutral. Finally, the functionalized MWCNTs were vacuum-dried at 80 °C for 12 h.

### 3.2. Synthesis of Bi_2_O_2_Se

Initially, 466 mg Bi_2_O_3_ powder (99%, Aladdin (Aladdin, Bay City, MI, USA)) and 395 mg Se powder (99%, Aladdin) were uniformly mixed through milling for 0.5 h. Subsequently, the mixture was transferred into a ceramic boat and calcined within a tube furnace at 600 °C under an Ar atmosphere for 1 h, with a heating rate of 5 °C min^−1^ and an airflow rate of 100 sccm. Finally, the prepared Bi_2_O_2_Se product was collected, milled, and stored in an argon-filled glove box for long-term storage, thereby mitigating potential surface oxidation.

### 3.3. Synthesis of Bi_2_O_2_Se–CNT Composites

The preparation processes of the three Bi_2_O_2_Se–CNT composites were analogous to that of pure Bi_2_O_2_Se, except that 70, 50, and 30 mg MWCNTs were added to the mixture of Bi_2_O_3_ and Se powders, respectively. For convenience, the resultant composites were named Bi_2_O_2_Se–CNT-1, Bi_2_O_2_Se–CNT-2, and Bi_2_O_2_Se–CNT-3, respectively. The raw materials in the prepared samples is shown in Table 1. Based on mass conservation principles, the theoretical CNT content was calculated to be 11.7%, 8.6%, and 5.4% in composites Bi_2_O_2_Se–CNT-1, Bi_2_O_2_S–CNT-2, and Bi_2_O_2_Se–CNT-3, respectively.

### 3.4. Material Characterization

The morphological features of the samples were examined using field-emission scanning electron microscopy (FESEM, JEOL JSM-IT800) operated at an accelerating voltage of 20 kV with a working distance of 9.3 mm. Prior to imaging, samples were sputter-coated with a 5 nm Au layer to enhance conductivity. Crystal phases were identified by X-ray diffraction (XRD, Bruker D8 ADVANCE) with Cu Kα radiation (*λ* = 1.5406 Å) at 40 kV and 40 mA. Data were collected in a 2*θ* range of 5–80° with a step size of 0.02° and a scan rate of 5°·min⁻¹. Raman spectroscopy was performed on a Horiba XploRA PLUS system equipped with a 532 nm laser (25% power, 50× objective) and a 600-groove·mm⁻¹ grating. Spectra were acquired over 100–1800 cm⁻¹ with a 1 cm⁻¹ resolution, and the system was calibrated using a silicon reference (520.7 cm⁻¹ peak). Surface chemical states were analyzed by X-ray photoelectron spectroscopy (XPS, Kratos AXIS SUPRA+) with a monochromatic Al Kα source (1486.6 eV). Charge correction was applied using the adventitious carbon C 1s peak (284.8 eV).

### 3.5. Electrochemical Measurement

The lithium storage properties of all samples were evaluated using CR2032 coin batteries assembled in an argon-filled glovebox (H_2_O/O_2_ < 0.1 ppm). Working electrodes were fabricated by coating a homogeneous slurry containing active material, carbon black, and polyvinylidene fluoride (PVDF) binder in a 70:20:10 wt% ratio onto copper foil and vacuum drying at 60 °C for 12 h. The slurry was prepared by dispersing components in N-methyl-2-pyrrolidone (NMP). The mass of the active materials deposited on the copper foils was about 1.0 mg cm^−2^. The batteries employed lithium foil as both counter and reference electrodes, with 1 M LiPF_6_ in EC/DMC (1:1 *v*/*v*) as the electrolyte and polypropylene membrane as the separator. Electrochemical measurements were conducted using a workstation (Metrohm AUTOLAB PGSTA302N) for cyclic voltammetry (CV, 0.1–3.0 V vs. Li⁺/Li) and electrochemical impedance spectroscopy (EIS, 100 kHz-0.01 Hz). Galvanostatic charge–discharge curves were recorded on a battery testing instrument (LAND CT2001A) at 0.1–3.0 A g^−1^ current densities.

## 4. Conclusions

In summary, a 2D/3D hybrid architecture of layered Bi_2_O_2_Se integrated with CNTs was synthesized via a high-temperature calcination strategy. The optimized Bi_2_O_2_Se–CNT-2 composite features a well-interconnected conductive network, where functionalized CNTs establish robust interfacial coupling with crystalline Bi_2_O_2_Se lamellae without compromising their structural integrity. This unique configuration synergizes the high-capacity multi-mechanism Li^+^ storage (intercalation, conversion, alloying) of layered Bi_2_O_2_Se with the 3D CNT framework, which ensures rapid electron transport and effective buffering against volume variations. Benefiting from these synergistic effects, the Bi_2_O_2_Se–CNT-2 anode achieves an exceptional initial discharge capacity of 1544.7 mA h g^−1^ at 0.1 A g^−1^, surpassing pristine Bi_2_O_2_Se (124.3 mA h g^−1^), while demonstrating superior rate performance (405.0 mA h g^−1^ at 2 A g^−1^) and cyclability (74.8% capacity retention after 250 cycles). Therefore, the rationally designed 2D/3D hybrid architecture in this work successfully achieves an optimal balance between high capacity and structural stability for LIBs anodes. This study demonstrates a universal strategy for harnessing the potential of 2D layered materials through their rational integration with 3D carbon-based frameworks, thereby advancing anode development for next-generation energy storage systems.

## Figures and Tables

**Table 1:** Composition raw materials for the prepared samples.

Samples	Bi_2_O_3_ Mass (g)	Se Mass (g)	MWCNTS Mass (g)
Bi_2_O_2_Se	466	395	0
Bi_2_O_2_Se–CNT-1	466	395	70
Bi_2_O_2_Se–CNT-2	466	395	50
Bi_2_O_2_Se–CNT-3	466	395	30

## Data Availability

Data are contained within the article and Supplementary Materials.

## Supplementary Materials

The following supporting information can be downloaded at https://www.mdpi.com/article/10.3390/molecules30081685/s1. Figure S1: FESEM comparison of Bi_2_O_2_Se–CNT-1, Bi_2_O_2_Se–CNT-2, and Bi_2_O_2_Se–CNT-3 at different magnifications; Figure S2: XPS survey spectra of Bi_2_O_2_Se–CNT-2; Figure S3. CV curves of Bi2O2Se for the initial three cycles at 0.2 mV s−1.; Figure S4. Galvanostatic charge–discharge curves of Bi_2_O_2_Se–CNT-2 at different current densities from 0.1 A g−1 to 2 A g−1; Figure S5. FESEM images of both Bi_2_O_2_Se–CNT-2 composite and pure Bi_2_O_2_Se electrodes after 250 cycles; Figure S6. Comparison of impedance of Bi_2_O_2_Se–CNT-1 (a), Bi_2_O_2_Se–CNT-2 (b), Bi_2_O_2_Se–CNT-3 (c), and pure Bi_2_O_2_Se (d) before cycle and 1st cycle; Table S1. Comparison of cycle performance between our Bi_2_O_2_Se–CNT-2 composite and other Bi_2_O_2_Se- or Bi_2_Se_3_-based anode materials for LIBs; Table S2. Values of *Rct*, *σ,* and *D* for different electrodes.
