# Supplementary material for: Rational Design of 2D/3D Bi2O2Se–CNT Hybrid Architectures for Synergistic Lithium Storage
Source: Molecules. 2025 Apr 9;30(8):1685. doi: 10.3390/molecules30081685 (PMC12029583; doi:10.3390/molecules30081685)
Supplement: Supplementary file 1 [file molecules-30-01685-s001.zip › molecules-3551119-supplementary.pdf]

Rational Design of 2D/3D Bi_2O_2Se–CNT Hybrid Architectures for Synergistic Lithium Storage

Duqiang Xin[1, 2*], Yue Zhang[1], Yeming He[1], Jiao Liu[1], Wenyuan Duan[1], Guoxiu Han[3], Qi Zhang[1] and Yuming Yang[1]

[1] *School of Electronic Information, Xijing University, Xi'an, 710123, People's Republic of China*

[2] *Shaanxi Engineering Research Center of Controllable Neutron Source, Xijing University, Xi'an, 710123, People's Republic of China*

[3] *School of Foreign Languages, Xijing University, Xi'an, 710123, People's Republic of China*

[*] Correspondence: xindq@foxmail.com (Duqiang Xin)

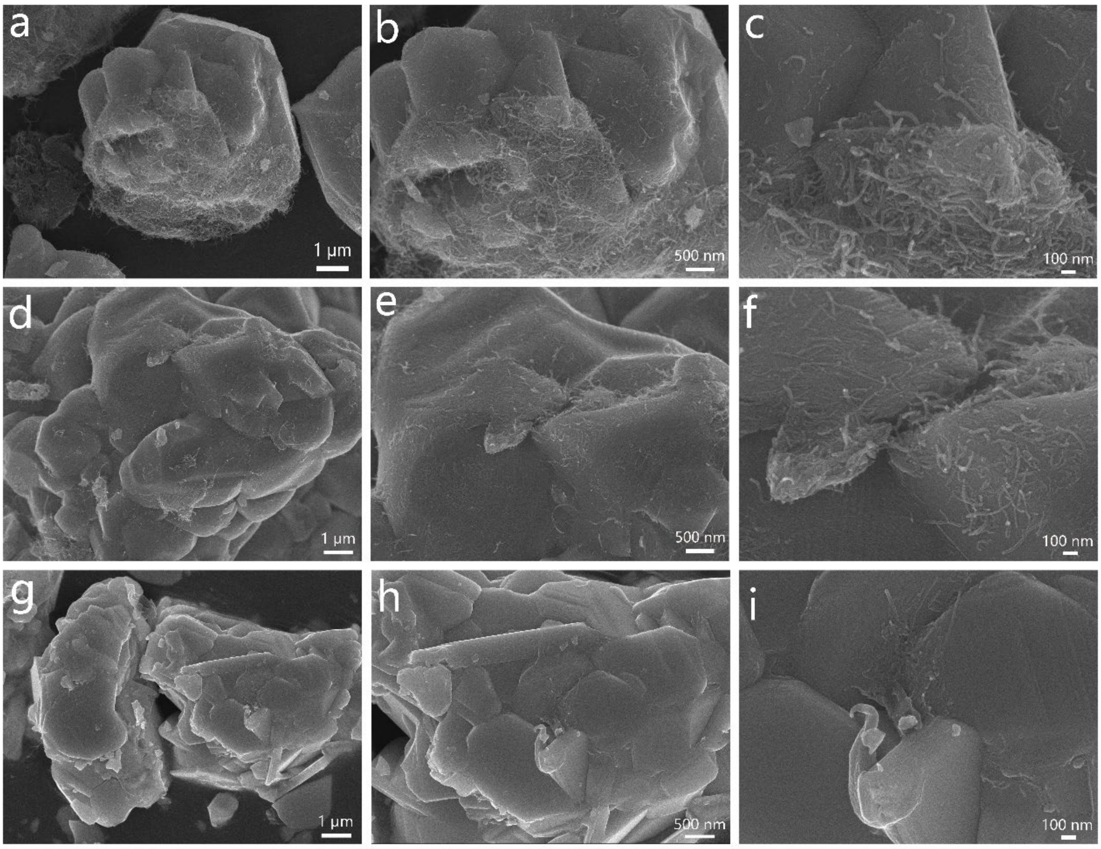

Figure S1. FESEM comparison of (a-c) Bi_2O_2Se–CNT-1, (d-f) Bi_2O_2Se–CNT-2, and (g–i) Bi_2O_2Se–CNT-3 at different magnifications.

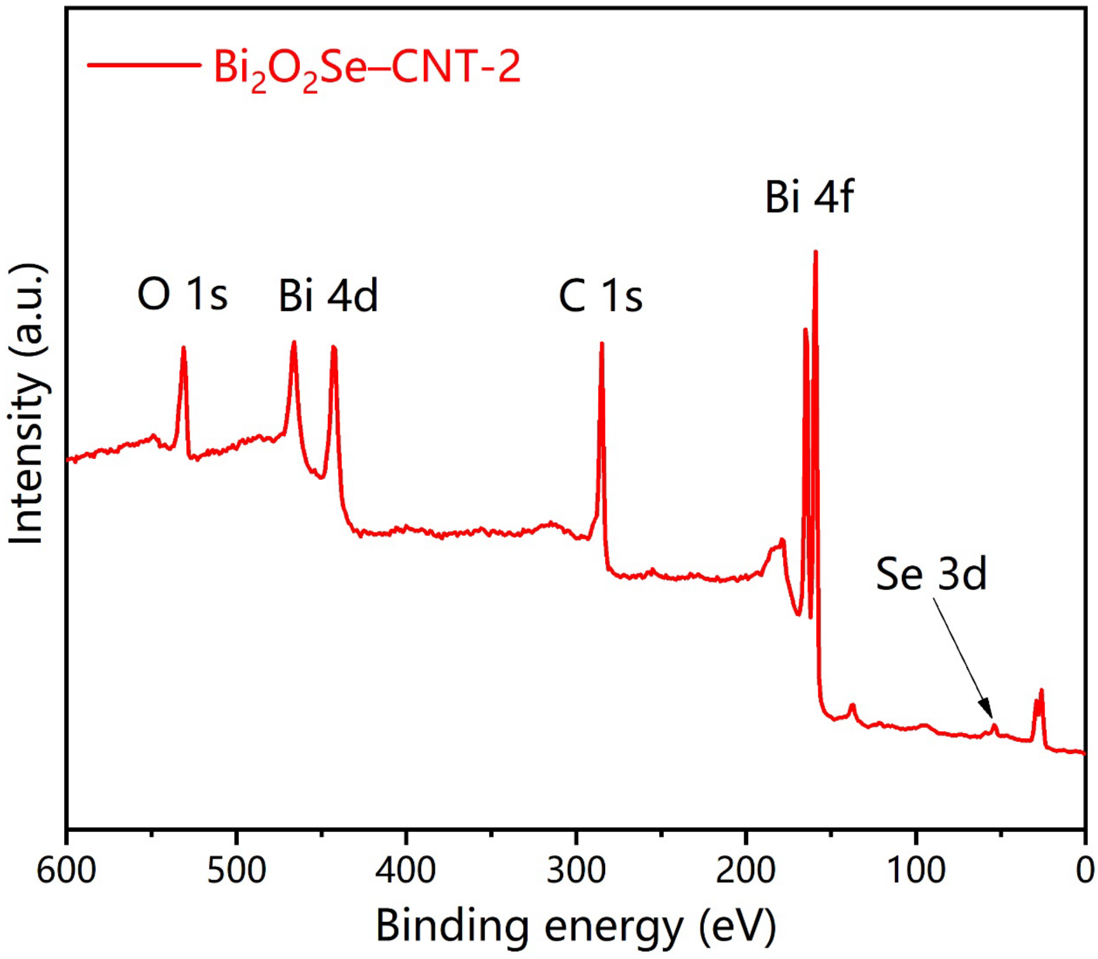

Figure S2. XPS survey spectra of Bi_2O_2Se–CNT-2.

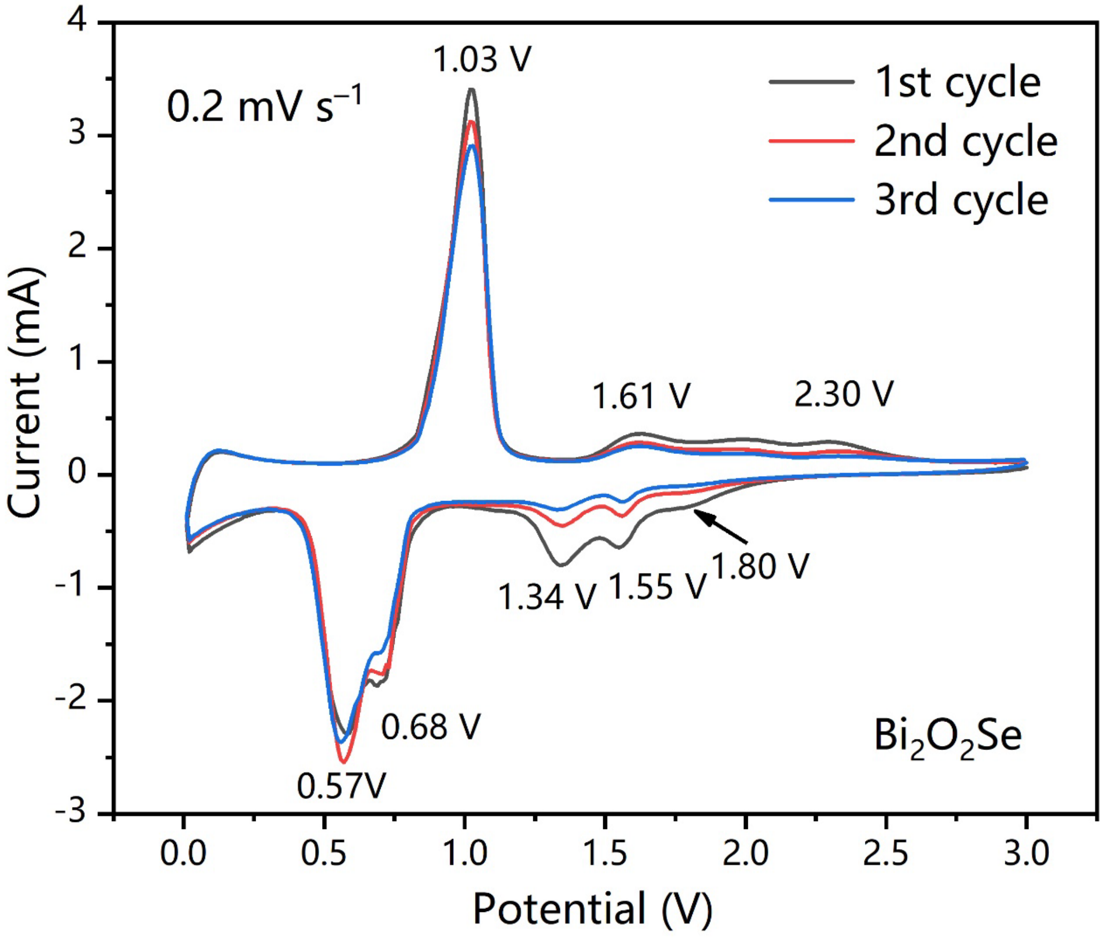

Figure S3. CV curves of Bi_2O_2Se for the initial three cycles at 0.2 mV s^{-1}.

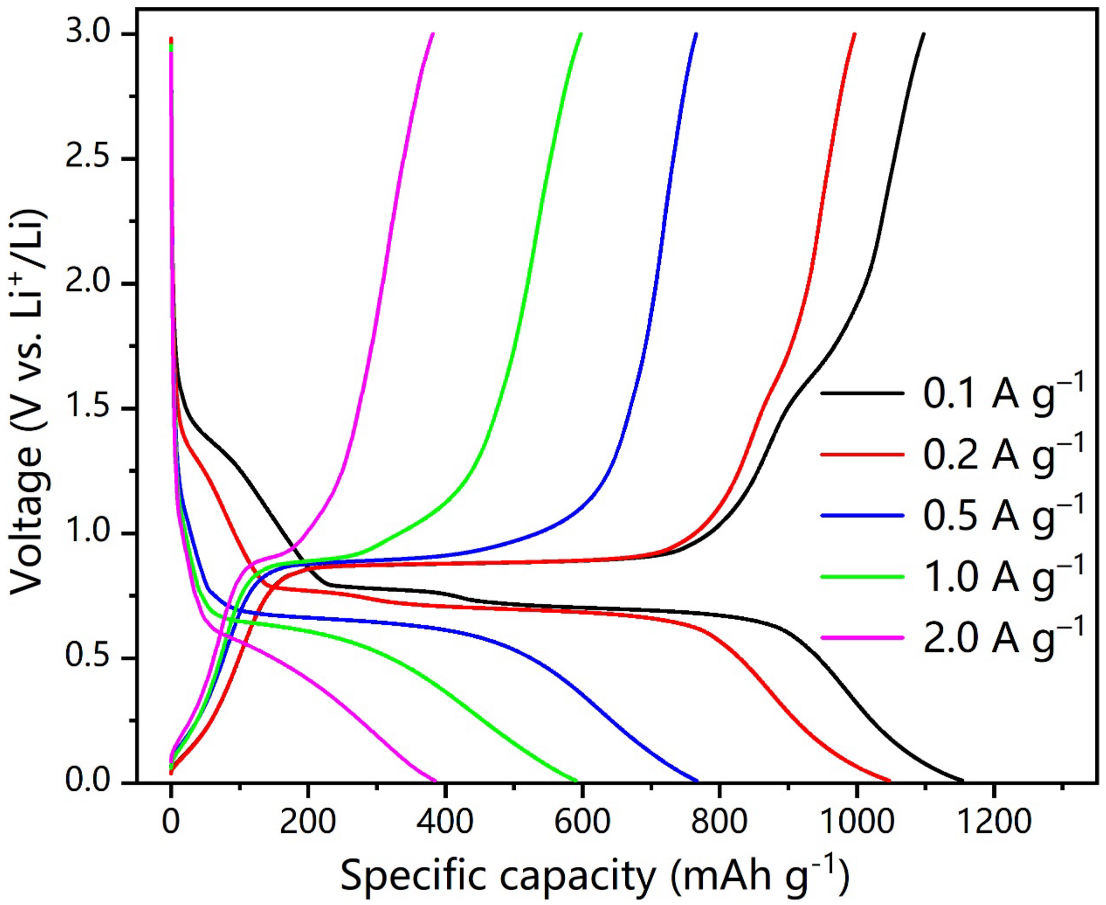

Figure S4. Galvanostatic charge/discharge curves of Bi_2O_2Se–CNT-2 at different current densities from 0.1 A g^{-1} to 2 A g^{-1}.

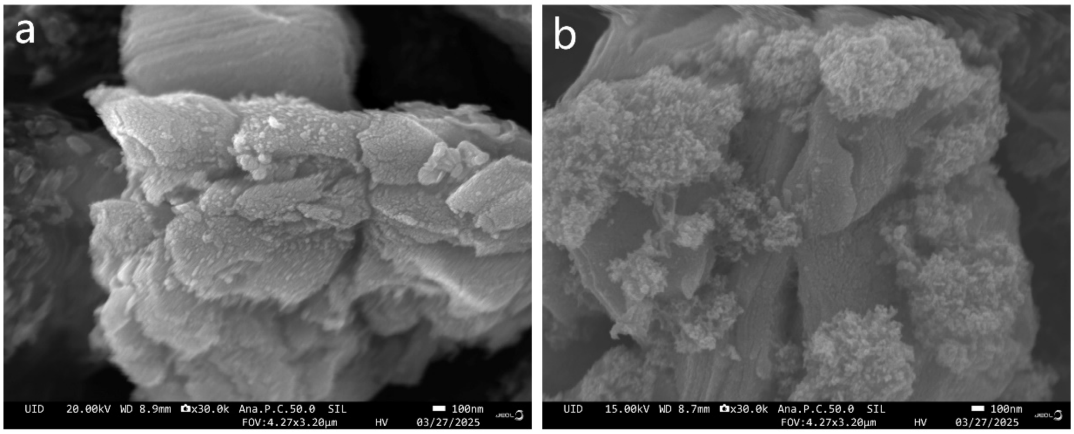

Figure S5. FESEM images of both Bi_2O_2Se–CNT-2 composite and pure Bi_2O_2Se electrodes after 250 cycles

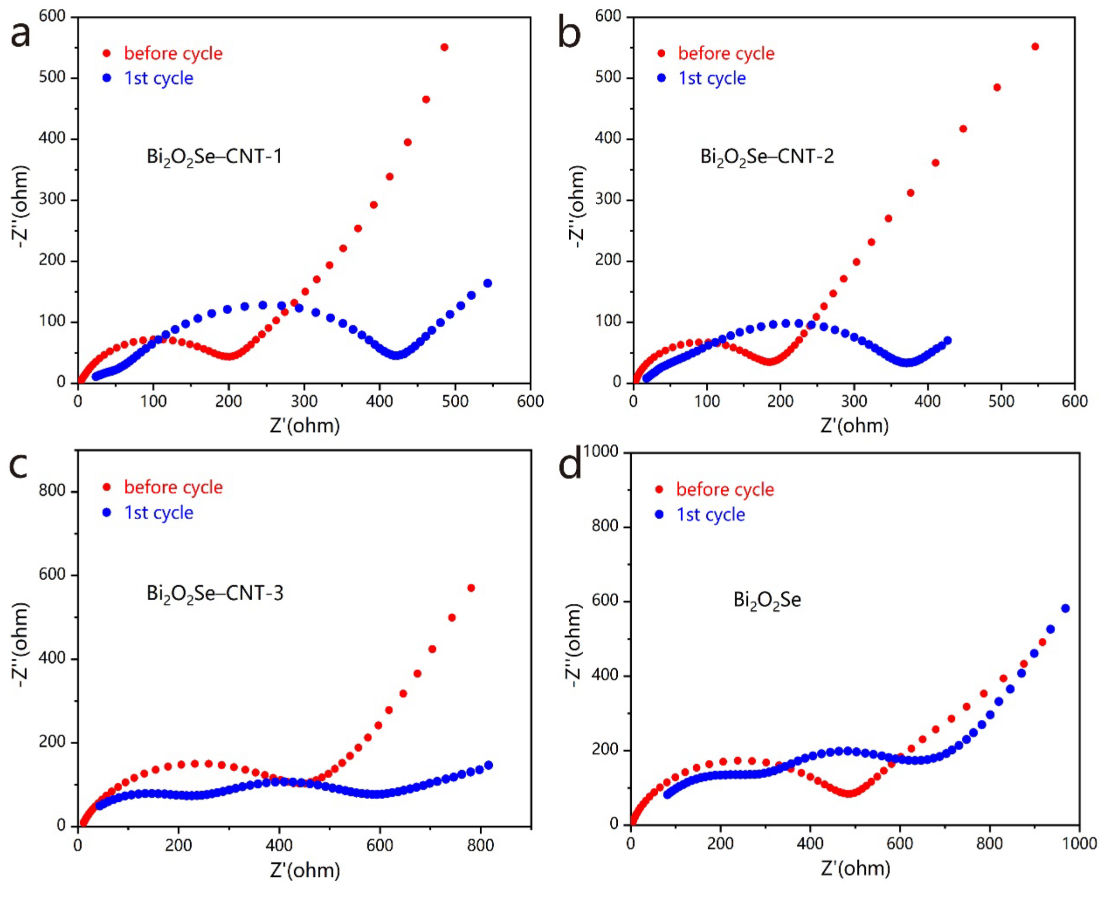

Figure S6. Comparison of impedance of Bi_2O_2Se–CNT-1 (a), Bi_2O_2Se–CNT-2 (b), Bi_2O_2Se–CNT-3 (c) and pure Bi_2O_2Se (d) before cycle and 1st cycle

Table S1. Comparison of cycle performance between our Bi_2O_2Se–CNT-2 composite and other Bi_2O_2Se or Bi_2Se_3 based anode materials for LIBs

Samples	Cycle number	Current density (A g^{-1})	Specific capacity (mAh g^{-1})	Ref.
Bi_2O_2Se/G	100	1.0	300	[1]
Bi_2Se_3@C	500	0.5	358	[2]
G-Bi_2Se_3/C	120	0.1	467.5	[3]
Bi_2Se_3-NFs@rGO	300	0.5	819	[4]
CNTs@C@Bi_2Se_3	300	1	243	[5]
Bi_2Se_3@NC	400	0.5	335.8	[6]
Bi_2Se_3/CDs	950	1	502	[7]
Bi_2Se_3/Mxene/SWCNT	900	10	320	[8]
**Bi_2O_2Se–CNT-2**	**250**	**1.0**	**450.4**	**This work**

Table S2. Values of R_{ct}, σ, and D for different electrodes

Electrode	R_{ct} (Ω)	σ (Ω $s^{-1/2}$)	D(m^2 s^{-1})
Bi_2O_2Se–CNT-1	206.5	402.3	1.98×10^{-17}
Bi_2O_2Se–CNT-2	172.2	301.2	3.55×10^{-17}
Bi_2O_2Se–CNT-3	428.7	313.1	3.28×10^{-17}
Bi_2O_2Se	439.9	403.2	1.97×10^{-17}

References

[1] Wu, Z.; Liang, G.; Wu, J.; Pang, W.K.; Yang, F.; Chen, L.; Johannessen, B.; Guo, Z. Synchrotron X-ray absorption spectroscopy and electrochemical study of Bi_2O_2Se electrode for lithium-/potassium-ion storage. Adv. Energy Mater. 2021, 11, 2100185.

[2] Dang, Z., Meng, W., Zuo, D., Li, D., Jiang, L., Fang, D. Synthesis of pomegranate-like Bi_2Se_3@C composite for high volume specific capacity lithium storage. Electrochimica Acta 2022, 425, 140752.

[3] He, B., Cunha, J., Hou, Z., Li, G., and Yin, H. 3D hierarchical self-supporting Bi_2Se_3-based anode for high-performance lithium/sodium-ion batteries. J. Colloid Interface Sci. 2023, 650, 857-864.

[4] Moon, J.H., Seong, H., Kim, G., Jin, Y., Nam, W., Yoo, H., Jung, T., Lee, K., Yang, M., Cho, S.Y., *et al.* Synthesis of Nanoflakes-like Bi_2Se_3@rGO composite and study on electrochemistry properties for high performance as the anode in lithium ion batteries. Appl. Surf. Sci. 2023, 638, 157976.

[5] Jin, R., Sun, M., and Li, G. CNTs@C@Bi_2Se_3 composite as an improved-performance anode for lithium ion batteries. Ceram. Int. 2017, 43, 17093-17099.

[6] Li, Z., Pan, H., Wei, W., Dong, A., Zhang, K., Lv, H., and He, X. Bismuth metal-organic frameworks derived bismuth selenide nanosheets/nitrogen-doped carbon hybrids as anodes for Li-ion batteries with improved cyclic performance. Ceram. Int. 2019, 45, 11861-11867.

[7] Wang, A., Hong, W., Li, L., Guo, R., Xiang, Y., Ye, Y., Zou, G., Hou, H., and Ji, X. Hierarchical bismuth composite for fast lithium storage: Carbon dots tuned interfacial interaction. Energy Storage Materials, 2022, 44, 145-155.

[8] Meija, R., Lazarenko, V., Rublova, Y., Felsharuk, A., Andzane, J., Gogotsi, O., Baginskiy, I., Zahorodna, V., Dutovs, A., Voikiva, V., *et al.* High-performance Bi_2Se_3/MXene/SWCNT heterostructures as binder-free anodes in lithium-ion batteries. Mater. Chem. Front. 2024, 8, 1651-1664.
